# Immunomodulatory therapies for COVID-19

**DOI:** 10.3389/fmed.2022.921452

**Published:** 2022-08-03

**Authors:** Poonam Mathur, Shyamasundaran Kottilil

**Affiliations:** Institute of Human Virology, University of Maryland School of Medicine, Baltimore, MD, United States

**Keywords:** severe COVID-19, viral infectious diseases, immunomodulatory therapies, COVID-19 pandemic, inpatient treatment

## Abstract

**Purpose:**

As COVID-19 disease progresses, the host inflammatory response contributes to hypoxemia and severe and critical illness. In these latter stages of disease, patients may benefit from immunomodulatory therapies to control the aberrant host inflammatory response. In this review, we provide an overview of these therapies and provide summaries of the studies that led to issuance of FDA Emergency Use Authorization or recommendation by the Infectious Diseases Society of America (IDSA).

**Materials and methods:**

We reviewed English-language studies, Emergency Use Authorizations (EUAs), and guidelines from March 2020 to present.

**Conclusion and relevance:**

There are several therapies with proposed benefit in severe and critical COVID-19 disease. Few have been issued FDA EUA or recommendation by the Infectious Diseases Society of America (IDSA). Physicians should be familiar with the evidence supporting use of these therapies and the patient populations most likely to benefit from each.

## Introduction

**FIGURE 1:**
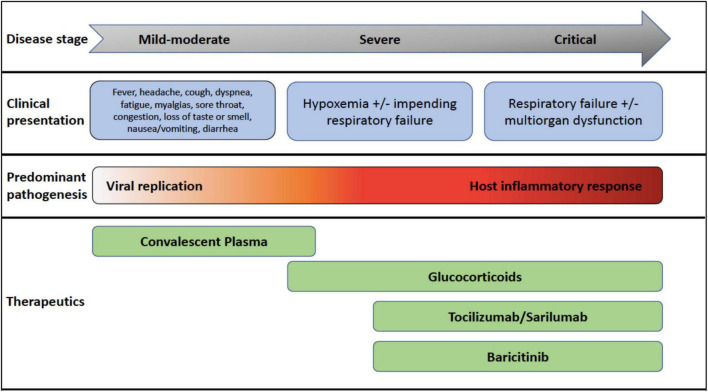
COVID-19 disease stages, clinical presentations, pathogenesis, and recommended therapeutics most beneficial at each disease stage.

The global pandemic caused by SARS-Coronavirus (CoV)-2 has exposed our vulnerability to the emergence of novel pathogens and the need to urgently expand our armamentarium of available antiviral therapeutics. It is clear now that COVID-19 has two phases, an initial mild-moderate disease primarily driven by viral replication and a severe-to-critical second phase dominated by host inflammatory response (Figure 1; 1). While vaccines have been shown to prevent and ameliorate COVID-19 disease, many nations are still reporting COVID-19-related deaths due to lack of immunizations and emergence of variants (2, 3). Mortality among infected individuals is driven by an aberrant host inflammatory response, and there is still an unmet need for optimizing care for severe and critically ill COVID-19 patients. In this review, we will explore the evidence that has accumulated from the use of immunomodulating therapies for COVID-19 disease over the last year. We will reference severity of illness as described in the IDSA guidelines for COVID-19 (4): mild to moderate (ambulatory), mild-to-moderate (hospitalized), severe (requiring oxygen supplementation), and critical (requiring ICU care).

## Materials and methods

We searched PubMed and the Cochrane databases for English-language studies published from March 1, 2020 to January 1, 2022 for randomized clinical trials (RCTs), meta-analyses, systematic reviews, and observational studies. We also manually reviewed and summarized IDSA guidelines and FDA Emergency Use Authorizations (EUAs) issued for COVID-19 therapeutics. Emphasis given to RCTs, meta-analyses, systematic reviews, EUAs, the IDSA guidelines, and to consideration of information of interest to a general medical readership.

## Discussion of therapies

**FIGURE 2:**
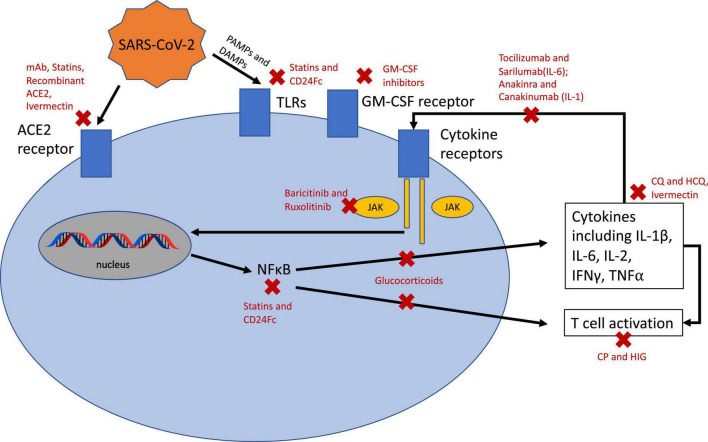
Mechanisms of action for therapeutics with potential benefit in SARS-CoV-2 infection. ACE2, angiotensin converting enzyme 2; TLRs, toll-like receptors; JAK, Janus kinase; NFκB, nuclear factor Kappa B; IL, interleukin; IFN, interferon; TNF, tumor necrosis factor; CP, convalescent plasma; HIG, hyperimmune globulin; CQ, chloroquine; HCQ, hydroxychloroquine; GM-CSF, granulocyote-monocyte colony stimulating factor.

A summary of the proposed mechanisms of action for the therapies discussed is shown in Figure 2. A summary of the therapies issued an FDA EUA or recommended by the IDSA is shown in Table 1.

**TABLE 1:** Immunomodulatory therapies recommended by the Infectious Diseases Society of America (IDSA) and/or FDA for COVID-19 treatment.

Therapeutic	Adult patient population	Dosing	Potential adverse reactions	Certainty of recommendation
Glucocorticoids (Dexamethasone preferred)	Hospitalized and/or severe COVID-19 disease.	Dexamethasone 6 mg IV or PO × 10 days or until discharge.	Hyperglycemia, neurological side effects (agitation/confusion), adrenal suppression, risk of bacterial or fungal infection	Moderate
Tocilizumab	Hospitalized with severe COVID-19 disease, elevated inflammatory markers, requiring supplemental oxygen, NIMV, IMV, or ECMO.	Weight < 30 kg: 12 mg/kg IV over 60 min. Weight > 30 kg: 8 mg/kg IV over 60 min. (Maximum dose 800 mg)	Increased risk of infection, gastrointestinal perforation (seen in non-COVID settings)	Low
Sarilumab	Hospitalized who meet criteria for tocilizumab, but it is not available.	400 mg IV over 60 min.	Increased risk of infection	Very Low
Baricitinib	Hospitalized with severe COVID-19 disease and elevated inflammatory markers, requiring supplemental oxygen, NIMV, IMV, or ECMO. Also indicated for use with remdesivir when corticosteroid contraindicated.	Baricitinib 4 mg PO daily × 14 days or until discharge.	Increased risk of infection, bowel perforation, thromboembolism, ischemic colitis, elevated transaminases, seizure	Moderate
Convalescent plasma (high-titer antibody)	Outpatient or hospitalized, with immunosuppressive disease or receiving immunosuppressive treatment, early in disease course.	NA	Circulatory overload, transfusion-associated lung injury, allergic transfusion reaction, thromboembolism	Low

Certainty of Recommendation Grades (based on data from clinical trials, and the risk of bias, inconsistency, indirectness, imprecision, and publication bias noted in the studies):

High: Based on data from clinical trials, the true effect lies close to that of the estimate of the effect.

Moderate: Based on data from clinical trials, the true effect is likely to be close to the estimate of the effect, but there is a possibility that it is substantially different.

Low Certainty: Based on data from clinical trials, the true effect may be substantially different form the estimate of the effect.

Very Low Certainty: Based on data from clinical trials, the true effect is likely to be substantially different from the estimate of the effect.

IV, intravenous; PO, oral; NIMV, non-invasive mechanical ventilation; IMV, invasive mechanical ventilation; ECMO, extracorporeal membrane oxygenation; NA, not applicable.

### Glucocorticoids

Corticosteroids are appealing agents due to their broad anti-inflammatory effects against cytokines such as Interleukin (IL)-1, IL-2, Tumor Necrosis Factor (TNF)-α, and IFN-gamma, which can lead to sepsis and respiratory failure (5). Early in the pandemic, there was hesitancy to use steroids due to prior studies showing steroids caused more harm than benefit in viral illnesses. For example, a 2019 meta-analysis and systematic review of more than 6,500 patients with influenza pneumonia showed that steroids increased the risk of mortality and secondary infection (6). In addition, steroids delayed viral clearance from the respiratory tract and increased adverse events in patients with SARS and MERS (7).

In July 2020, preliminary data from the RECOVERY (8) study was published, an open label trial randomizing patients hospitalized with COVID-19. The primary outcome was 28-day mortality. A total of, 2,104 patients received oral or IV dexamethasone 6 mg daily for up to 10 days, and 4,321 received usual care. Significantly more patients who received usual care died compared to those in the dexamethasone group, and reduction in mortality correlated with the level of respiratory support required. In patients who required invasive mechanical ventilation or non-invasive oxygen support, the mortality rates were significantly lower, but not in those who did not require respiratory support at randomization. These findings may seem contradictory to the aforementioned studies, but the benefit seen in that requiring oxygen supplementation is likely due to the pathogenesis in the severe and critical stages of SARS-CoV-2 infection (Figure 1). Therefore, steroids are more beneficial in the latter, hyperinflammatory stages (7). Currently, the IDSA recommends use of glucocorticoids in patients who are hypoxic from COVID-19 (i.e., severe or critical illness) (4).

### Cytokine inhibitors

COVID-19 disease is associated with a rapid innate immune response, causing a surge in cytokines that cause leads to pulmonary and vascular dysfunction (9). Cytokines such as IL-1α, IL-1β, IL-6, and TNF-α are produced in excess and potentiate pathological processes such as pneumonitis and vascular thrombosis (10). Cytokine release is stimulated by recognition of viral RNA by innate pattern recognition receptors, such as toll-like receptors (TLRs). Recognition of single-stranded RNA viruses *via* TLRs promotes activation of Nuclear Factor κB and transcription of pro-inflammatory cytokines such as IL-1β and IL-6 (11). IL-1 has been found to impair endothelial barrier function and capillary leak (a significant issue in COVID-19 pneumonitis), and the IL-6-JAK-STAT3 axis has been found to be activated in severe COVID-19 disease (12). IL-6 levels are associated with severity of COVID-19 disease, independent of age or sex (13).

Early in the pandemic, tocilizumab, a monoclonal antibody which antagonizes the IL-6 receptor, was of interest for use in COVID-19 disease. However, the initial studies had varying results (14–20). Later RCTs (17, 21, 22), including pre-prints, demonstrated improvement in clinical outcomes, including mortality, with the use of tocilizumab. The populations included in these trials were heterogeneous, ranging from patients not requiring oxygen but with an inflammatory phenotype, to those requiring invasive mechanical ventilation. Most of the data to support the use of tocilizumab comes from the RECOVERY (23) trial. This was a randomized, open-label trial of 4,116 patients with oxygen saturation ≤ 92% on room air and CRP ≥ 75 mg/L who received standard of care (including steroids) vs. standard of care and tocilizumab. The primary outcome was 28-day mortality. There was a significant benefit in survival with tocilizumab, which was augmented with concomitant steroid use. Among patients not already intubated, tocilizumab prevented progression to invasive ventilation or death.

The REMAP CAP (24) study also showed benefit of IL-6 antagonists for critically ill COVID-19 patients. In this study, 803 patients were randomized to receive placebo, tocilizumab, or sarilumab (another IL-6 antagonist). The primary outcome was respiratory and cardiovascular organ support-free days. Tocilizumab and sarilumab increased the number of organ support-free days and survival. In the RECOVERY and REMAP CAP studies, the IL-6 antagonists were administered within 48 h of hospitalization, indicating that treatment with these agents early in the severe COVID-19 stage provides the most benefit. As of February 2021, the IDSA recommends use of tocilizumab in addition to standard of care (i.e., steroids) for those patients with elevated markers of inflammation and progressive severe or critical COVID-19 disease (4).

The IDSA recommends that sarilumab can be used in place of tocilizumab, if it is not available (4). However, in an international trial, double-blind RCT of 45 hospitals (25), sarilumab was not effective in improving time to clinical improvement among hospitalized patients receiving sarilumab 400 or 200 mg compared to placebo. This data on sarilumab suggests that it may not be as effective as tocilizumab for use in severe COVID-19 disease. More trials evaluating sarilumab’s efficacy to improve mortality are needed.

Inhibition of IL-1 is also of interest since the cytokine enhances neutrophil activity, which can become uncontrolled and lead to respiratory failure (26). The beneficial effects of IL-1 inhibition with the use of the monoclonal antibody anakinra have been summarized in case reports of patients with macrophage activation syndrome (27) and severe COVID-19 pneumonia (28, 29). Early in the pandemic, prospective, retrospective, and open-label studies in COVID-19 disease did not demonstrate clinical benefit from IL-1 antagonists (30–32). The use of canakinumab, an IL-1β inhibitor, was investigated in the CAN-COVID trial, which was randomized, double-blind, and placebo- controlled. An interim analysis did not find any increase in likelihood of survival without invasive mechanical ventilation at day 29 compared to placebo (33). Currently, no IL-1 antagonists are approved for COVID-19 treatment.

### Monoclonal antibodies

Early in the pandemic, monoclonal antibodies (mAbs) were manufactured as a means of passive immunity while awaiting vaccine development. mAbs differ from convalescent plasma (CP, discussed below), in that CP consists of polyclonal antibodies from someone previously infected with SARS-CoV-2; mAbs have singular activity against a pre-determined target. The mAbs manufactured for use in COVID-19 disease mainly target the spike protein of SARS-CoV-2, thus blocking viral entry. Products with FDA authorization for COVID-19 disease include bamalanivab/etesevimab, casirivimab/imdevimab, and sotrovimab (34). Tixagevimab/cilgavimab has also been authorized for pre-exposure prophylaxis in immunocompromised patients or those patients who are not expected to mount adequate antibody response to vaccination. With the emergence of the omicron variant, however, bamlanivimab/etesivimab, casirivimab/imdevimab, and sotrovimab have significantly reduced neutralizing activity, so the use of these monoclonal antibodies is no longer recommended (35, 36). Tixagevimab/cilgavimab retains activity against the omicron variant (37). In February 2022, the FDA issued an EUA for the mAb bebtelovimab, which has been shown to retain activity against the omicron variant (38).

With the emergence of new SARS-CoV-2 variants, there is always potential that mAbs will lose neutralizing activity since their utility is as antiviral agents. In contrast, though host T cell responses may be mitigated by viral epitope changes, immunomodulators are less likely succumb to changes in these epitopes. Even with new SARS-CoV-2 variants, immunomodulators continue to boost cellular and humoral immunity, retaining efficacy for treating severe COVID-19 disease (39).

### Janus-associated kinase inhibitors

Several cytokine responses in COVID-19 employ the JAK axis for intracellular signaling. The IL-6-JAK-STAT3 axis is activated in COVID-19 and regulates a myriad of cellular functions. Inhibiting the JAK pathway may reduce the deleterious effects of COVID-19 (40) by reducing cytokine production. There are several JAK inhibitors approved for various indications in the United States; baricitinib is the only JAK inhibitor to have an FDA EUA for use in hospitalized patients with COVID-19 disease. Data to support the EUA in severe COVID-19 disease comes from the ACTT-2 trial (41), in which patients with moderate to severe disease were randomized to receive baricitinib 4 mg PO daily with remdesivir vs. placebo with remdesivir. The combination of baricitinib and remdesivir showed a decreased trend toward mortality (4.7 vs. 7.1%; RR: 0.65; 95% CI 0.39, 1.09). In patients with severe COVID-19 disease on supplemental oxygen, the benefit was enhanced: the baricitinib group was more likely to experience clinical recovery (69.3 vs. 59.7%; RR 1.29; 95% CI 1.00, 1.66). The use of a JAK inhibitor did not increase the number of serious adverse events; in fact, there were fewer adverse events in the intervention arm.

In addition, the COV-BARRIER (42) study evaluated the efficacy of baricitinib for severe COVID-19 disease in hospitalized patients. Subjects were randomized to receive baricitinib or placebo for 14 days in addition to standard of care (including antivirals and steroids). There was a significant reduction in all-cause mortality at days 28 and 60 in the baricitinib group compared to placebo. Therefore, baricitinib (4, 43) is indicated for hospitalized patients with severe COVID-19 disease who have elevated inflammatory markers and/or cannot receive corticosteroids due to a contraindication. In the latter scenario, baricitinib is recommended in combination with the antiviral remdesivir rather than remdesivir alone. Most of the benefit is seen in patients who are not yet mechanically ventilated.

Ruxolitinib is another JAK inhibitor used in several disease states due to its ability to alter aberrant host immune responses. A multicenter, single-blind, and RCT in China (44) investigated the use of ruxolitinib in patients with severe COVID-19 disease, but did not find any benefit in time to clinical improvement. There are several ongoing clinical trials investigating the use of ruxolitinib in COVID-19 (40). However, future clinical trials will need to identify what, if any, advantage ruxolitinib has over baricitinib and steroids.

### Convalescent plasma and hyperimmune immunoglobulin

Convalescent plasma (CP) and HIG therapies have been used during influenza, Ebola, SARS-CoV, (5) and MERS outbreaks (45) with reduction in mortality and hospital stay duration, possibly because these antibodies can control viremia (45). CP refers to plasma collected after disease recovery and HIG is similar to intravenous immune globulin (IVIG), but collected from donors with high antibody titers against a specific infection. Both HIG and CP provide passive antibody therapy and may blunt or prevent response to infection (46). Both are also associated with adverse events, including transfusion-associated reactions and thrombosis (5). In addition, there is a theoretical risk of antibody-dependent enhancement (ADE) (47), where previously-developed antibodies that are transfused worsen clinical severity as a result of infection with a different viral type, however, there have been no reports of ADE with use of CP in SARS-CoV or MERS infections (46).

Early in the pandemic, CP was an attractive option, since no purified monoclonal antibodies were available. Small anecdotal studies demonstrated benefit from CP (48), but larger studies showed equivocal and even discouraging data. A multi-site RCT in Argentina (49) of 333 hospitalized patients with COVID-19 found no significant difference in mortality 30 days after intervention with CP or placebo. A Cochrane database systematic review (50) included 20 studies (of which one was an RCT that was stopped early) assessing the safety and efficacy of HIG and CP use. In these studies, most subjects received CP (5,211 of 5,443) and the rest received HIG. The review found low-certainty evidence for safety of CP for COVID-19. However, a retrospective study based on the US National Death Registry (51) showed promising results for CP based on antibody titer. Among 3,082 patients included in the analysis, there was a mortality benefit associated with high antibody titer CP (signal to cutoff ratio > 18.45) compared to low titer (ratio < 4.62). Mortality benefit was seen in those who were not on mechanical ventilation at the time of CP transfusion. A multicenter, double-blind, RCT of 1,181 outpatients with COVID-19 found that high-titer CP reduced hospitalizations by 50%, regardless of risk factors for disease, progression, or vaccine status. Based on this study, in March, 2021, the FDA issued a EUA for high-titer CP for hospitalized patients with COVID-19 early in the disease course (i.e., prior to mechanical ventilation) and with impaired humoral immunity. However, a randomized trial from Italy (TSUNAMI) of 487 patients with COVID-19 pneumonia found that high-titer CP did not significantly reduce the rate of mechanical ventilation or death 30 days after CP compared to standard therapy (remdesivir, steroids, and heparin). These results were consistent across subgroups of age, sex, race, comorbidities, and use of concomitant therapy (52). One reason for the discrepancy among the studies investigating high-titer CP may be the timing of administration. In the study that demonstrated CP reduced hospitalization, there was a median 4 days from SARS-CoV-2 onset to CP administration, whereas in the latter study, the median time of CP administration after onset of disease was 7.7 days, and it has been suggested that antibody-based therapies are more effective in early stages of the disease. Currently, the IDSA recommends against CP for hospitalized patients (4).

### Anti-malarials

The antimalarials chloroquine (CQ) and hydroxychloroquine (HCQ) have expanded use beyond as anti-infectives. They inhibit cytokine production (5) and block viral replication by blocking viral fusion (53). An *in vitro* study early in the pandemic showed CQ reduced viral copies of SARS-CoV-2 (54). A systematic review and meta-analysis of non-RCTs (55) suggested HCQ may have some benefit in COVID-19 disease, but this was limited to reduction in radiologic progression of lung disease. These trials and an open-label study also suggested safety and efficacy of HCQ in combination with azithromycin (56), but this was not supported by findings in a non-human primate study (57). A systematic review found that RCTs of HCQ and CQ use in COVID-19 have selection, performance, and detection biases (58). A more recent systematic review (59) of publications with low-to-moderate risk of bias suggests that CQ and HCQ alone or in combination with azithromycin may not be effective and in fact, associated with harm. Therefore, neither the FDA nor IDSA (4) recommend the use of CQ or HCQ for treatment of hospitalized patients with COVID-19.

### Anti-parasitics

Previous studies have suggested that ivermectin has anti-inflammatory effects (4). For these reasons, ivermectin was a therapeutic of interest for severe COVID-19 disease. In 2020, *in vitro* studies showed that that ivermectin decreased SARS-CoV-2 viral replication (60, 61). However, *in vitro* activity in human tissues and plasma requires drug concentrations higher than those achieved with approved doses of the drug (62). Some RCTs show benefit in clinical outcomes and mortality, however there is a high risk of bias due to unsuccessful randomization into treatment and placebo arms, heterogeneity of outcomes, and lack of blinding in these studies (4). In addition, there have been significant side effects and drug-drug interactions associated with the use of ivermectin (63). Currently, neither the FDA nor IDSA (4) recommend ivermectin for COVID-19 treatment.

### Granulocyte-macrophage colony-stimulating factor inhibitors

Granulocyte-macrophage colony-stimulating factor (GM-CSF) is a myelopoietic growth factor and pro-inflammatory cytokine secreted by many cells including macrophages and T cells. Its pro-inflammatory properties can result in tissue damage; GM-CSF is thought to be a key driver of lung inflammation and possibly, ARDS, in severe COVID-19. Therefore, anti-GM-CSF monoclonal antibodies may mitigate inflammation and prevent lung tissue damage in severe COVID-19 disease. Gimsilumab, lenzilumab, namilumab, and otilimab are GM-CSF inhibitors, blocking the interaction of GM-CSF with its cell surface receptor. Another GM-CSF inhibitor is mavrilimumab, which targets the alpha subunit of the GM-CSF receptor, blocking downstream, intracellular signaling. To date, clinical trials have not demonstrated clear benefit of GM-CSF inhibitors in COVID-19. The only published RCT demonstrating benefit is LIVE-AIR, a double-blind RCT of lenzilumab, which showed significant improvement in the primary endpoint of ventilatory-free survival through Day 28 among those who received the GM-CSF inhibitors (64). However, a large, double-blind, randomized trial of otilimab (65), in which the primary endpoint was survival free from respiratory failure at Day 28, and a small, double-blind, randomized trial of mavrilimumab, in which the primary endpoint was proportion alive and off supplemental oxygen at Day 14 (66), did not show a survival benefit for the GM-CSF inhibitors compared to placebo. Of note, the studies investigating lenzilumab and mavrilimumab included patients on room air or oxygen by nasal cannula only, whereas the otilimab study did not include these patients and only had patients with high-flow oxygen, non-invasive or invasive ventilation. Last, the recently published BREATHE trial (67) investigated the use of gimsilumab in hospitalized patients with COVID-19 across the United States. In this double-blind, placebo-controlled, RCT, the primary endpoint was all-cause mortality rate at Day 43. Gimsilumab did not improve mortality compared to placebo, or other key clinical outcomes, including ventilator-free survival rate. Currently, neither the FDA nor IDSA endorse the use of GM-CSF inhibitors for severe COVID-19 disease.

### Angiotensin converting enzyme 2 agonists

SARS-CoV-2 enters cells *via* its spike protein, which binds to the ACE2 receptor on target cells (5). Blocking viral binding to the ACE2 receptor is a potential therapeutic target. A double-blind, randomized, placebo-controlled phase 2 clinical trial using recombinant human ACE2, APN01, is currently ongoing (NCT04335136) (68). APN01 essentially mimics ACE2 *in vivo*, blocking SARS-CoV-2 from binding to cells. Study enrollment has completed and efficacy data from the trial is expected soon. In the interim, a case report published in September 2020 provides the first promising data for APN01 in COVID-19 disease (69).

There was controversy surrounding the continued use of ACE inhibitors (ACEIs) and angiotensin-receptor blockers (ARBs) to treat hypertension early in the pandemic. The concern was that SARS-CoV-2 would downregulate the ACE2 receptor (by binding), allowing angiotensin II, unopposed by ACE2, to increase oxidative stress, inflammation, and fibrosis. However, an RCT of 659 hospitalized patients with mild-to-moderate COVID-19 showed no significant difference in the mean number of days alive and out of the hospital whether or not ACEIs and ARBs were continued (70). In addition, a case-control study of over 6,000 patients with severe COVID-19 also suggested that ACEIs or ARBs did not affect disease progression in COVID-19 (71). Currently, the American College of Cardiology and American Heart Association recommend continuing ACEI and ARB therapy in patients with COVID-19 (72).

### Statins

Besides lowering lipids, statins have anti-inflammatory effects, including inhibition of the TLR-NFκB pathway *in vitro* (73). *In vivo*, statins have been shown to lower CRP, adding to the evidence that statins modulate inflammation. Some studies during the 2009 H1N1 pandemic suggested statins played a role in reducing disease severity and mortality among hospitalized patients (73).

Statins may alter the disease course in COVID-19 (73, 74). First, statins may interfere with coronavirus entry *via* alteration of ACE2 and CD147 expression. Second, statins may protect against thrombosis in COVID-19 by preventing endothelial barrier damage and by impairing transcription of pro-coagulant factors. Third, statins may decrease the number of lipid rafts (small membrane domains enriched in lipids) available for virus assembly and proliferation. Fourth, statins may increase cell autophagy, allowing for viral particles to be degraded intracellularly.

Several studies have suggested that statins reduce COVID-19 disease severity (75–77). In a meta-analysis (78) of 8,990 patients with COVID-19, statins reduced the risk of severe or fatal disease from SARS-CoV-2 [HR = 0.70, 95% CI (0.53–0.94)]. However, no RCT is available to guide the addition of statins as standard of care treatment for COVID-19. Also, despite potential benefits, there has been hesitancy in using statins for COVID-19 due to drug-drug interactions (*via* CYP3A4), and toxicity in the setting of liver and/or renal failure. Currently, there is no FDA or IDSA recommendation to prescribe statins solely for the treatment of COVID-19.

### Fusion proteins

Damage- and pathogen-associated molecular patterns (DAMPs and PAMPs) play a critical role in initiating cellular inflammatory responses such as that seen in COVID-19. Inflammatory responses can be suppressed by exploiting innate checkpoint mechanisms, such as Sialic acid-binding immunoglobulin-like lectins (Siglecs), which have inhibitory motifs and regions that suppress *in vitro* and *in vivo* DAMP-mediated innate inflammatory responses (79). CD24Fc, a recombinant fusion protein of CD24 (found on many cells in the body, including hematopoietic cells, immature neuronal cells, and epithelial cells) (80), and the Fc portions of human IgG1, has been shown to inhibit the host inflammatory response to DAMPs. It does so by (1) binding to DAMPs, eliminating the stimulus for inflammatory cascades, and (2) binding to Siglecs, activating intracellular inhibitory tyrosine-based motifs, which ultimately results in inhibition of transcription factors such as NFkB. *In vivo*, CD24Fc ameliorates graft-versus-host disease, an unfortunate potential consequence of allogeneic hematopoietic stem cell transplantation, mediated by proinflammatory cytokines, DAMPs, and PAMPs and activation of antigen-presenting cells (79, 81). In Chinese rhesus macaques infected with simian immunodeficiency virus (SIV), CD24Fc protects against progression to AIDS (82), presumably by mitigating SIV-induced cytokine production and inflammation (83). In addition, CD24Fc protects against SIV-mediated pneumocyte death, a phenotype associated with ARDS (84).

Since ARDS is a prominent feature of critical COVID-19 disease, CD24Fc was investigated in the SAC-COVID trial (NCT04317040), a multi-site phase III trial of hospitalized patients with severe or critical COVID-19 (85). Participants were randomized to receive one dose of CD24Fc 480 mg or placebo at the time of randomization, in addition to standard of care (i.e., steroids and/or remdesivir). The primary outcome was the time to clinical improvement over 28 days. A planned interim analysis of 192 subjects showed an increased clinical improvement rate of 81.8% in the CD24Fc arm compared to 66.3% for the placebo arm. Median time to clinical improvement was 6.0 days (95% CI 5.0–8.0) in the CD24Fc group versus 10.0 days (7.0–15.0) in the placebo group [hazard ratio (HR) 1.61, 95% CI 1.16–2.23; log-rank *p* = 0.0028, which crossed the prespecified efficacy boundary (α = 0.0147)]. The final enrollment included 234 subjects. Median time to clinical improvement was 6.0 days (95% CI 5.0–9.0) in the CD24Fc group versus 10.5 days (7.0–15.0) in the placebo group (HR 1.40, 95% CI 1.02–1.92; log-rank *p* = 0.037). The proportion of participants with disease progression within 28 days was 19% (22 of 116) in the CD24Fc group versus 31% (36 of 118) in the placebo group (HR 0.56, 95% CI 0.33–0.95; unadjusted *p* = 0.031). The incidences of adverse events and serious adverse events were similar in both groups. No treatment-related adverse events were observed. There was also a numerical trend toward reduction in respiratory failure and mortality with use of CD24Fc (22 vs. 28%), but these results were not significant, as the study was not powered for these outcomes. This study suggests that CD24Fc can be used as an adjunct to standard of care therapies for COVID-19 to augment clinical improvement and possibly reduce respiratory failure and mortality. CD24Fc may be the ideal compound given its mechanism of action that preserves antigen-specific immune response while preventing DAMP/PAMP mediated aberrant immune response. This molecule offers hope as a selective immunomodulator, striking a balance between immunosuppression and maintained host antiviral response.

## Conclusion

In order to combat a pandemic caused by a novel virus, therapies which target immune dysregulation brought about by SARS-CoV-2 infection are urgently needed and should be further investigated. A common theme in regulation of the detrimental effects of respiratory viruses like SARS-CoV-2 is finding methods to dampen the exacerbated inflammatory effects of the virus which lead to respiratory failure and severe sepsis and/or shock. An extraordinary number of randomized trials investigating agents for COVID-19 treatment are underway, focusing on a multitude of treatment modalities. Many of the aforementioned agents focus on targeting the innate immune system in order to block production of pro-inflammatory cytokines and have received endorsement from the IDSA, as well as EUA issuance by the FDA for use in COVID-19 disease. The most studied and widely-used immunomodulator for COVID-19 disease is steroids. However, glucocorticoids have multiple effects on the host immune system (86), including non-specific immunosuppression. In addition, glucocorticoids are associated with side effects, such as unmasking of diabetes mellitus and hypertension. Hence, there is a need for steroid-sparing therapy for COVID-19. Ideal agents are those that selectively dampen an undesirable host inflammatory response without altering T-cell and monocyte-mediated antiviral activity. Therefore, innovative therapies, such as CD24Fc, offer a better safety prolife than steroids, and may provide protection against severe COVID-19 disease by mitigating the host inflammatory response without causing broad immunosuppression. CD24Fc’s unique mechanisms warrant adding this agent to our arsenal of therapeutics, as we continue combatting the COVID-19 pandemic.

## Author contributions

PM wrote the first draft of the manuscript. Both authors contributed to conception, design, and revision of the manuscript and read and approved the submitted version.
